# Downregulation of miR-637 promotes vascular smooth muscle cell proliferation and migration via regulation of insulin-like growth factor-2

**DOI:** 10.1186/s11658-020-00222-z

**Published:** 2020-05-07

**Authors:** Ning Yang, Bo Dong, Yanqiu Song, Yang Li, Lu Kou, Jingyu Yang, Qin Qin

**Affiliations:** Department of Cardiology, Tianjin Chest hospital, Taierzhuang South Road No.261, Jinnan District, Tianjin, 300222 China

**Keywords:** miR-637, IGF-2, VSMC, Atherosclerosis

## Abstract

**Background:**

Dysregulation of the proliferation and migration of vascular smooth muscle cells (VSMCs) is a crucial cause of atherosclerosis. MiR-637 exerts an antiproliferative effect on multiple human cells. Its impact on atherosclerosis remains largely unexplored.

**Methods:**

Real-time PCR was used to determine miR-637 expression in samples from atherosclerosis patients and animal models. Its expression in VSMC dysfunction models (induced by ox-LDL) was also measured. The proliferation and migration of VSMCs were respectively tested using CCK-8 and Transwell assays, and apoptosis was measured using flow cytometry. The Targetscan database was used to predict the target genes of miR-637. Interaction between miR-637 and the potential target gene was validated via real-time PCR, western blotting and a luciferase reporter assay.

**Results:**

MiR-637 expression was significantly lower in atherosclerosis patient and animal model samples. It also decreased in a dose- and time-dependent manner in animal models with ox-LDL-induced atherosclerosis. Transfection with miR-637 mimics suppressed the proliferation and migration of VSMCs while promoting apoptosis, while transfection with miR-637 inhibitors had the opposite effects. We also validated that insulin-like growth factor-2 (IGF-2), a crucial factor in the pathogenesis of atherosclerosis, serves as a target gene for miR-637.

**Conclusion:**

MiR-637 targeting IGF-2 contributes to atherosclerosis inhibition and could be a potential target for this disease.

## Introduction

Cardiovascular disease (CVD) is a leading cause of death and disability worldwide [1]. Atherosclerosis is a major cause of many CVDs [2, 3]. Recent studies show that atherosclerosis is a complicated process in which monocytes, foam cells and endothelial cells play different roles [4–6]. Furthermore, increasing evidence indicates that vascular smooth muscle cells (VSMCs) have a crucial role in the progression of atherosclerosis [7].

During the development of atherosclerosis, VSMCs change from contraction- to synthetic-type cells, which leads to abnormal changes in their proliferation and migration. Dysfunction of VSMCs facilitates atherosclerotic plaque formation in the early stages and prevents fiber cap rupture in the advanced stage [8–11]. The molecular mechanisms of VSMC dysfunction in atherosclerosis remain poorly elucidated.

MicroRNA (miRNA) is a type of non-coding RNA of about 20 nucleotides in length. MiRNAs are involved in multiple biological processes, including cell proliferation and apoptosis. A number of studies have verified that miRNAs have crucial roles in the behaviors of VSMCs during the process of atherosclerosis through their regulation of multiple signaling pathways. For instance, miR-135b-5p and miR-499a-3p facilitate the proliferation of VSMCs [12]. MiR-148a and miR-152 can reduce the expression of DNMT1 and facilitate the deposition of lipids in foam cells [13]. MiR-126 can suppress the PI3K/Akt/mTOR signaling pathway to alleviate endothelial cell injury [14].

MiR-637 has been found to play a role in various human diseases. It can restrain the proliferation of hepatoma cells by downregulating AKT1 [15] and modulate the malignant behavior of papillary thyroid carcinoma cells [16]. Its specific role and regulatory mechanisms in atherosclerosis remain unclear.

Insulin-like growth factor 2 (IGF-2) is a protein hormone that has a verified and pivotal role in various human diseases [17]. For instance, it has a diagnostic value in Silver-Russell syndrome and is closely related to the catch-up growth of low-birth-weight infants [18, 19]. Accumulating evidence confirms that IGF-2 can facilitate atherosclerosis by accelerating the proliferation and migration of VSMCs [20, 21].

Some miRNAs have been validated to regulate IGF-2. For example, in renal cell carcinoma, miR-615 exerts an inhibitory effect on IGF-2 expression [22]. In the tumorigenesis of breast cancer, miR-100 expression decreases, which upregulates IGF-2 to facilitate the cancer progression [23]. The regulatory mechanism of IGF-2 in atherosclerosis remains poorly understood.

The TargetScan database predicts that IGF-2 is a potential target of miR-637. Here, we aimed to explore the impact of the miR-637–IGF-2 axis on atherosclerosis. This is expected to help clarify the molecular mechanisms of atherosclerosis and provide novel therapeutic targets.

## Methods

### Clinical samples

The subjects were 57 patients presenting with atherosclerosis plaques at Tianjin Chest Hospital between January 1 and December 31, 2018, and 57 healthy volunteers recruited as controls. Blood samples (3 ml) were collected from each subject. After the blood was centrifuged at 3000 g for 10 min, the plasma (no blood cells) was collected and stored at − 80 °C. All patients and volunteers signed informed consent and the research protocol was approved by the Ethics Committee of Tianjin Chest Hospital.

### Animal model

The animal experiment was approved by the Animal Ethics Committee of Tianjin Chest Hospital. This experiment involved 60 C57BL/6 8-week old mice from the Department of Laboratory Animal Science of Peking University Medical School. Of these, 50 were apolipoprotein E-knockout mice (ApoE^−/−^ mice) fed with high-fat diet for 12 weeks to induce atherosclerosis. The remaining 10 were wild-type mice fed a normal diet and used as the empty control.

For the experiment, 40 ApoE^−/−^ mice were divided into four groups of 10: the miR-637 agomir negative control (AG-NC), miR-637 agomir (AG), miR-637 antagomir negative control (AN- NC) and miR-637 antagomir (AN) groups. One week after the mice had adapted to their environment, miR-637 agomir or miR-637 antagomir were dissolved into 0.2 ml saline at a dose of 20 mg/kg body weight/day, and respectively injected into the AG and AN group mice through the tail vein once every 2 weeks. After the end of the experimental period, the mice were intraperitoneally injected with 20% uratan at a dose of 7 ml/kg body weight as a general anesthetic. When the muscle tension of the mice was weakened and the breathing was accelerated, blood was carefully collected and stored in a 5 ml EP tube. Then, the blood samples were centrifuged at 3000 g for 10 min at 4 °C for 5 min to obtain the plasma. In addition, the common carotid artery of the mice was isolated under aseptic conditions. After being washed with PBS, the intima was gently scraped off and the outer membrane was removed. The vascular smooth muscle layer was retained for subsequent experiments.

### Cell culture and transfection

Human VSMCs from the ATCC were cultured in RPMI-1640 containing 10% fetal bovine serum (FBS), 1% penicillin and 1% streptomycin (Gibco) at 37 °C and in 5% CO_2_. They were treated with different concentrations of ox-LDL (0, 25, 50 and 100 mg/l) for 24 h or with 100 mg/l ox-LDL for different times (0, 12, 24, 48 h). VSMCs in the log phase were selected for transfection. MiR-637 mimics and inhibitors were transfected using Lipofectamine 2000 (Invitrogen). Forty-eight hours later, the transfection efficiency was validated using real-time PCR.

### RNA extraction and quantitative real-time PCR analysis

To confirm that the plasma samples were hemolysis-free, their hemoglobin levels were measured using spectrophotometry (Thermo Fisher Scientific) and quantified with the formula CHb = 1.58A_415_–0.95A_450_–2.91A_700_ at the threshold sensitivity of 0.01 mg/ml. All the plasma samples included in the study contained ≤0.2 mg/ml hemoglobin.

Then, real-time PCR was used to determine the relative expression of miR-637 and IGF-2 mRNA. Total RNA was extracted from atherosclerosis patient plasma, healthy volunteer plasma, C57BL/6 mouse plasma and human VSMCs with TRIzol reagent (Invitrogen) according to the manufacturer’s instructions. To detect the relative expression of IGF-2 mRNA, reverse transcription was conducted using MMLV transcriptase (Invitrogen) to generate cDNA. To detect the relative expression of miR-637, reverse transcription was performed with a TaqMan MicroRNA Reverse Transcription Kit (Thermo Fisher Scientific). Quantitative real-time PCR was performed on the ABI 7500 Real-Time PCR System (Applied Biosystems) with SYBR premix EX TAQ II kit (TaKaRa) according to the manufacturer’s instructions. GAPDH and U6 were used as internal controls. The relative expressions were determined using the 2^-ΔΔCt^ method. In this study, primer sequences were constructed with the aid of Primer Premier 6.0 and are shown in Table 1.

**Table 1:** Primer sequences for real-time PCR

Name	Primer sequences
miR-637	Forward: 5′-AGCCCACACACTACAGGCA-3′
	Reverse: 5′-GCACAAAAGCAGTACGACCT-3′
IGF-2	Forward: 5′-CTTGGACTTTGAGTCAAATTGG-3′
	Reverse: 5′-GGTCGTGCCAATTACATTTCA-3′
U6	Forward: 5′-CTCGCTTCGGCAGCACATATACT-3′
	Reverse: 5′-ACGCTTCACGAATTTGCGTGTC-3′
GAPDH	Forward: 5′-GCAAGTTCAACGGCACAG-3′
	Reverse: 5′-GCCAGTAGACTCCACGACAT-3′

### CCK-8 assay

VSMCs at the log phase were selected and seeded in a 96-well plate (5 × 10^3^ cells/well) with 3 replicate wells for each sample type. Ten microliter CCK-8 solution (Hubei Baiaosi Bioscience) was added to each well. The OD value was measured at 450 nm using a microplate reader (Bio-Rad Laboratories).

### Apoptosis assay

The cells were seeded into a 6-well plate. When the cells had grown to 60–70% confluence, the medium was discarded. The plate was washed twice with PBS solution, and cells were trypsinized with 0.25% trypsin. Then the cells were collected in an EP tube and centrifuged at 1000 r/min and 4 °C for 5 min. The supernatant was removed and the cells were resuspended with 500 μl binding buffer. An FITC Annexin V cell apoptosis assay kit (Ruibo) was used to stain cells for 30 min at room temperature. After that, the apoptosis level was determined using a flow cytometer (Becton Dickinson).

### Transwell assay

The cells were serum-starved for 24 h and then trypsinized. The medium was discarded, the cells were washed twice in PBS solution, and then resuspended in serum-free medium containing BSA to adjust the cell density to 2 × 10^5^ cells/ml. Afterwards, 200 μl of cell suspension was added to a Transwell chamber (8 μm pore size; Corning), and the lower chamber was supplemented with RPMI-1640 containing 20% FBS. Following 24 h of culture at 37 °C and in 5% CO_2_, the culture medium in the upper chamber was discarded and the cells that had not passed through the membrane in the upper chamber were carefully wiped with cotton swabs. The upper chamber was then washed twice with PBS. Residual cells were fixed with 4% paraformaldehyde for 15 min and stained in 0.1% crystal violet for 30 min. Five fields were randomly selected under the microscope for counting.

### Western blotting

The cells were lysed with RIPA solution (Beyotime Biotechnology) containing the protease inhibitor PMSF and the total protein was extracted. The protein concentration was determined using the BCA method. Equivalent amounts of protein were separated via 10% SDS-PAGE and transferred onto nitrocellulose membrane. After being blocked with 5% skim milk, the membrane was incubated with primary antibody at room temperature for 1 h. The membrane was then washed with TBST solution, and incubated with secondary antibody at room temperature for 1 h, followed by washing with TBST solution. After the addition of ECL solution (Hubei Baiaosi Bioscience), color development was carried out, and the protein bands were quantitatively analyzed using Image J.

### Dual luciferase reporter assay

StarBase and TargetScan were used to predict the target sequence of miR-637. Wild-type (WT) and mutant-type (MUT) IGF-2 were subcloned into pGL3 vector (Promega) and transfected into VSMCs. The cells were then plated in 24-well plates with 1 × 10^5^ cells/well. Then, luciferase activity was determined with the dual luciferase system (Promega). MiR-637 mimics were transfected into VSMCs using wild or mutant vector. Luciferase activity was measured 48 h after the transfection.

### Statistical analysis

Statistical analysis was performed using SPSS 22.0. The measurement data are presented as means ± standard deviation. The independent samples *t* test was performed for the comparison between the two groups. The difference was statistically significant at *p* < 0.05.

## Results

### MiR-637 was abnormally expressed during the development of atherosclerosis

**Fig. 1:**
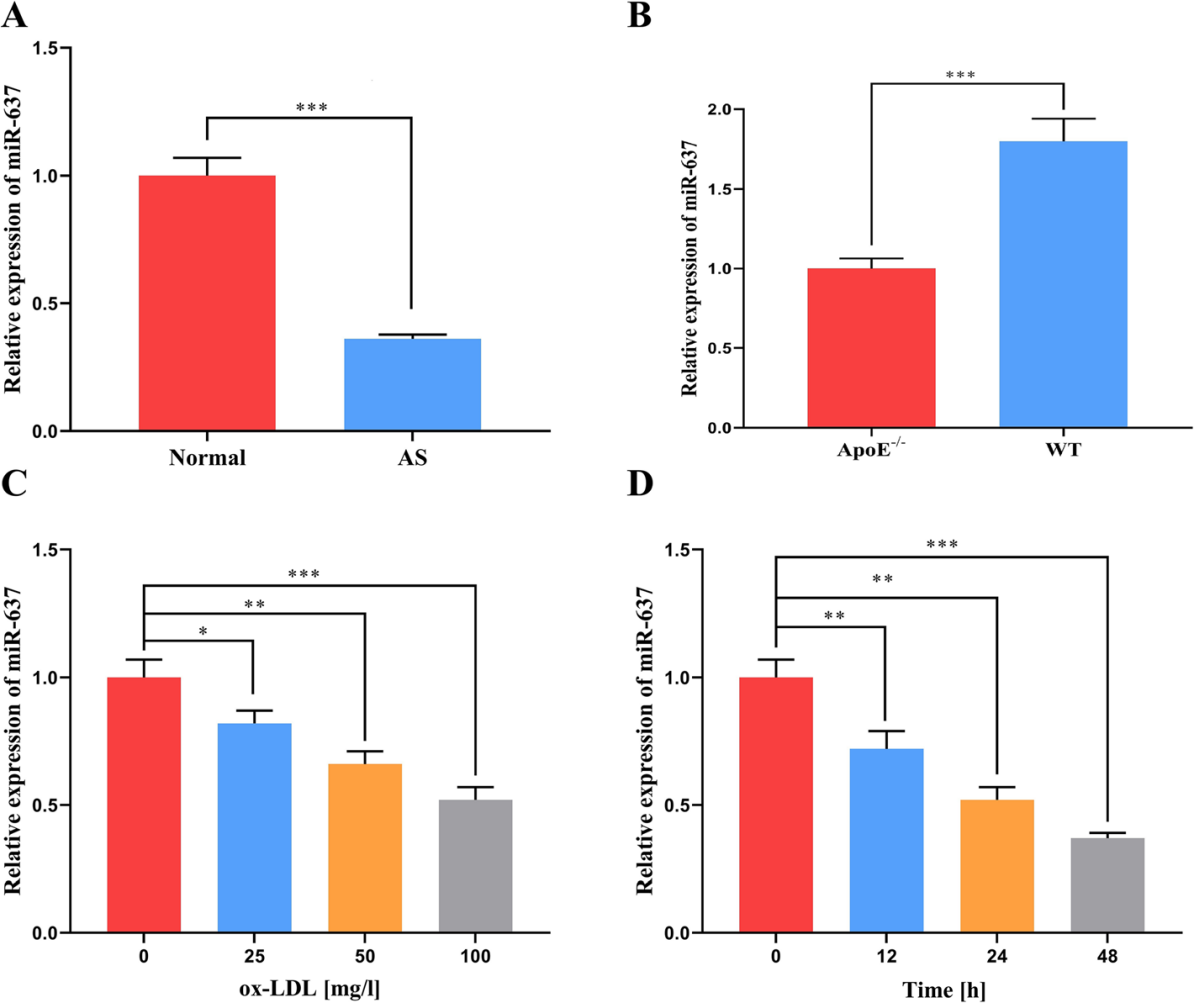
miR-637 is downregulated in atherosclerosis. **a** and **b** – The expression levels of miR-637 in human plasma (**a**) and mouse plasma (**b**) were determined using real-time PCR. **c** and **d** – The expression level of miR-637 in VSMCs treated with ox-LDL was determined using real-time PCR. *, ** and *** respectively indicate *p* < 0.05, *p* < 0.01 and *p* < 0.001

As shown, miR-637 expression was significantly downregulated in the plasma of the atherosclerosis patients (Fig. 1a). The expression of miR-637 in the plasma of ApoE^−/−^ C57BL/6 mice on the high-fat diet decreased significantly compared with the level for other groups (Fig. 1b). Finally, the expression of miR-637 in VSMCs treated with ox-LDL was also significantly lower, having been inhibited in both time- and dose-dependent manners (Fig. 1c and d).

### MiR-637 impeded the proliferation and migration of VSMCs

**Fig. 2:**
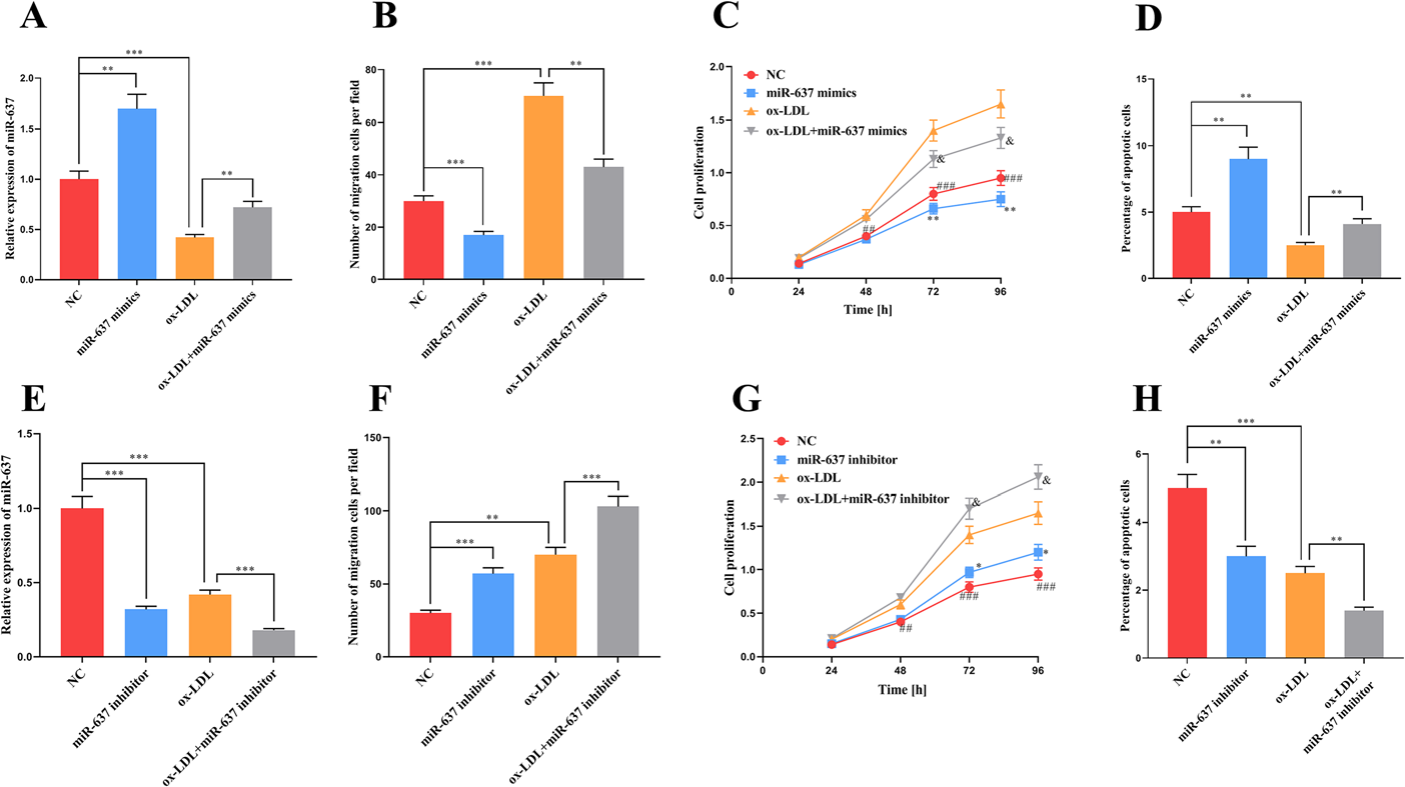
The effects of miR-637 on proliferation, migration and apoptosis of VSMCs. **a** – MiR-637 mimics were successfully transfected into VSMCs and VSMCs treated with 100 mg/l ox-LDL for 24 h. **b** – The migration of VSMCs was tested using a Transwell assay after transfection with miR-637 mimics. **c** – A CCK-8 assay was used to determine the effect of transfection with miR-637 mimics on the proliferation of VSMCs. **d** – The apoptosis rate of VSMCs was determined after transfection with miR-637 mimics. **e** – MiR-637 inhibitors were successfully transfected into VSMCs and VSMCs treated with 100 mg/l ox-LDL for 24 h. **f** – The migration of VSMCs was tested using a Transwell assay after transfection with miR-637 inhibitors. **g** – A CCK8 assay was used to determine the effect of transfection with miR-637 inhibitors on the proliferation of VSMCs. **h** – The apoptosis rate of VSMCs was determined after transfection with miR-637 inhibitors. *, ** and *** respectively indicate *p* < 0.05, *p* < 0.01 and *p* < 0.001. In Fig. C, * indicates *p* < 0.5, NC group VS miR-637 mimics group; ^&^ indicates *p* < 0.5, ox-LDL group VS ox-LDL + miR-637 mimics group; ^###^ indicates *p* < 0.001, NC group VS ox-LDL group. In Fig. G, * indicates *p* < 0.05, NC group VS miR-637 inhibitor group; ^&^ indicates *p* < 0.05, ox-LDL group VS ox-LDL + miR-637 inhibitor group; ^###^ indicates *p* < 0.001, ox-LDL group VS NC group

We successfully transfected miR-637 mimics and inhibitors into VSMCs and examined the cell phenotypes (Fig. 2a). The migration and proliferation of VSMCs was significantly inhibited after mimic transfection (Fig. 2b and c). MiR-637 mimics also increased the apoptosis of VSMCs (Fig. 2d). Conversely, inhibitors promoted migration and proliferation but inhibited apoptosis (Fig. 2e through h).

### MiR-637 targeted IGF-2

**Fig. 3:**
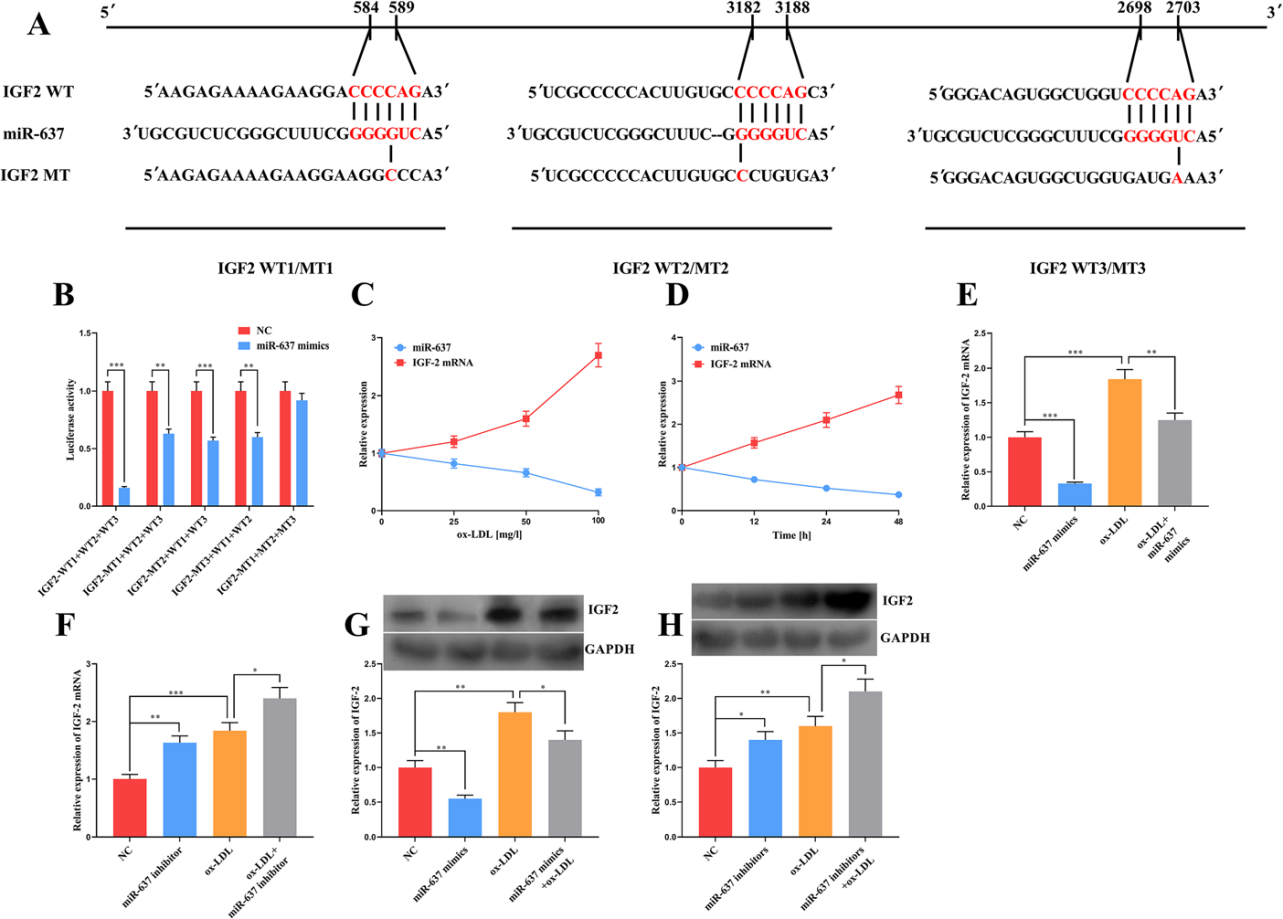
The interaction between miR-637 and IGF-2. **a** – TargetScan database prediction of the binding sites between IGF-2 and miR-637. **b** – The targeted relationship between miR-637 and IGF-2 was verified using a dual luciferase reporter gene assay. **c** and **d** – The expressions of miR-637 and IGF-2 mRNA showed different trends depending on the ox-LDL concentrations and treatment time. **e** – The expression of IGF-2 mRNA was determined using real-time PCR after treatment with miR-637 mimics, 100 mg/l ox-LDL or miR-637 mimics+ 100 mg/l ox-LDL. **f** – The expression of IGF-2 mRNA was determined using real-time PCR after treatment with miR-637 inhibitors, 100 mg/l ox-LDL or miR-637 inhibitors+ 100 mg/l ox-LDL. **g** – The expression of IGF-2 was determined using western blotting after treatment with miR-637 mimics, 100 mg/l ox-LDL or miR-637 mimics+ 100 mg/l ox-LDL. **h** – The expression level of IGF-2 was determined using western blotting after treatment with miR-637 inhibitors, 100 mg/l ox-LDL or miR-637 inhibitors+ 100 mg/l ox-LDL. *, ** and *** respectively indicate *p* < 0.05, *p* < 0.01 and *p* < 0.001

Through the TargetScan database, we found that IGF-2 was one of the targets for miR-637 (Fig. 3a). The existence of binding sites was then confirmed using the dual luciferase reporter assay (Fig. 3b). Moreover, the expression of miR-637 decreased and that of IGF-2 mRNA increased with the increase in ox-LDL concentration or treatment time (Fig. 3c and d). Additionally, it was confirmed that miR-637 mimics blocked the expression of IGF-2 mRNA and protein, while miR-637 inhibitor had the opposite effects (Fig. 3e through h). These results indicate that IGF-2 is a target gene of miR-637.

### IGF-2 reversed both the inhibitory effect of miR-637 on the proliferation and migration of VSMCs and the promotional effect on apoptosis

**Fig. 4:**
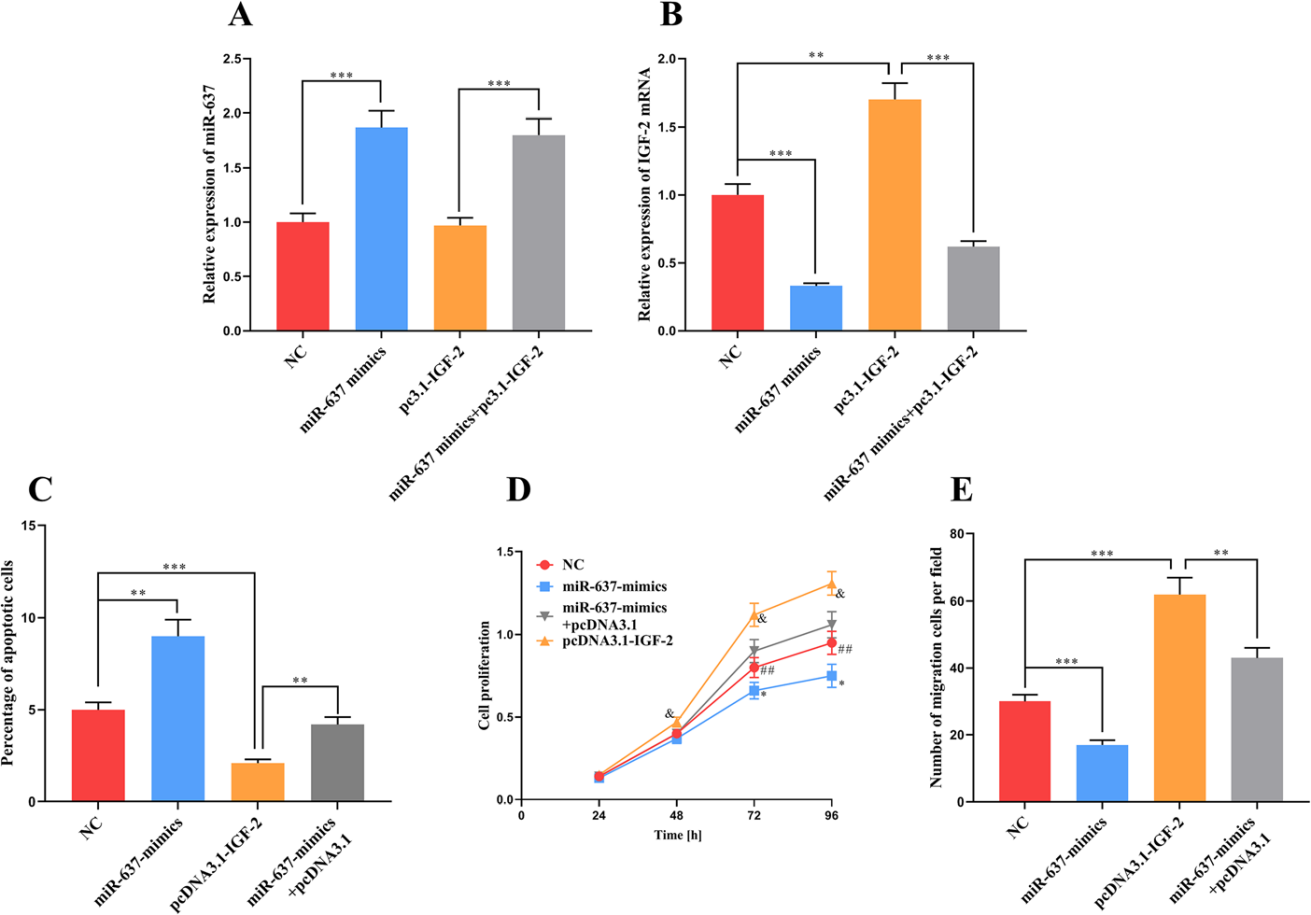
IGF-2 reverses the effect of miR-637 on the proliferation, migration and apoptosis of VSMCs. **a** and **b** – real-time PCR was carried out to determine the expressions of miR-637 (**a**) and IGF-2 mRNA (**b**) in VSMCs after transfection with miR-637 mimics, pcDNC3.1-IGF-2 or miR-637 mimics +pcDNA3.1-IGF-2. **c** – Flow cytometry was carried out to monitor the apoptosis of VSMCs in each group. **d** – A CCK8 assay was done to determine the proliferation of VSMCs in each group. **e** – A Transwell assay was performed to determine the migration of VSMCs in each group. *, **, *** respectively indicate *p* < 0.05, *p* < 0.01, *p* < 0.001. In Fig. D, * indicates *p* < 0.05, NC group VS miR-637 mimics group; ^##^ indicates *p* < 0.01, pcDNA3.1-IGF-2 group VS NC group; ^&^ indicates *p* < 0.05, pcDNA3.1-IGF-2 group VS miR-637 + pcDNA-IGF-2 group

To confirm the interaction between IGF-2 and miR-637, we successfully transfected miR-637 mimics, pcDNA3.1-IGF-2 and miR-637 mimics with pcDNA3.1-IGF-2 into VSMCs with or without ox-LDL treatment. The expression of IGF-2 mRNA was obviously suppressed by miR-637 mimics, but pcDNA3.1-IGF-2 had no impact on miR-637 expression (Fig. 4a and b), suggesting a unidirectional effect of miR-637 on IGF-2.

In the apoptosis experiment, transfection with miR-637 mimics promoted the apoptosis of VSMCs, while pcDNA3.1-IGF-2 reversed this effect (Fig. 4c). Transfection with miR-637 mimics suppressed the proliferation of VSMCs and pcDNA3.1-IGF-2 reversed this effect (Fig. 4d). The results of the Transwell migration assay indicated that transfection with miR-637 mimics impeded the migration of VSMCs and that pcDNA3.1-IGF-2 reversed this inhibitory effect. Moreover, the migration of VSMCs transfected with pcDNA3.1-IGF-2 was significantly facilitated (Fig. 4e). These results imply that the functions of miR-637 in VSMCs are partly mediated by IGF-2.

### The influence of miR-637 on the expressions of IGF-2 mRNA and protein in a mouse model of atherosclerosis

**Fig. 5:**
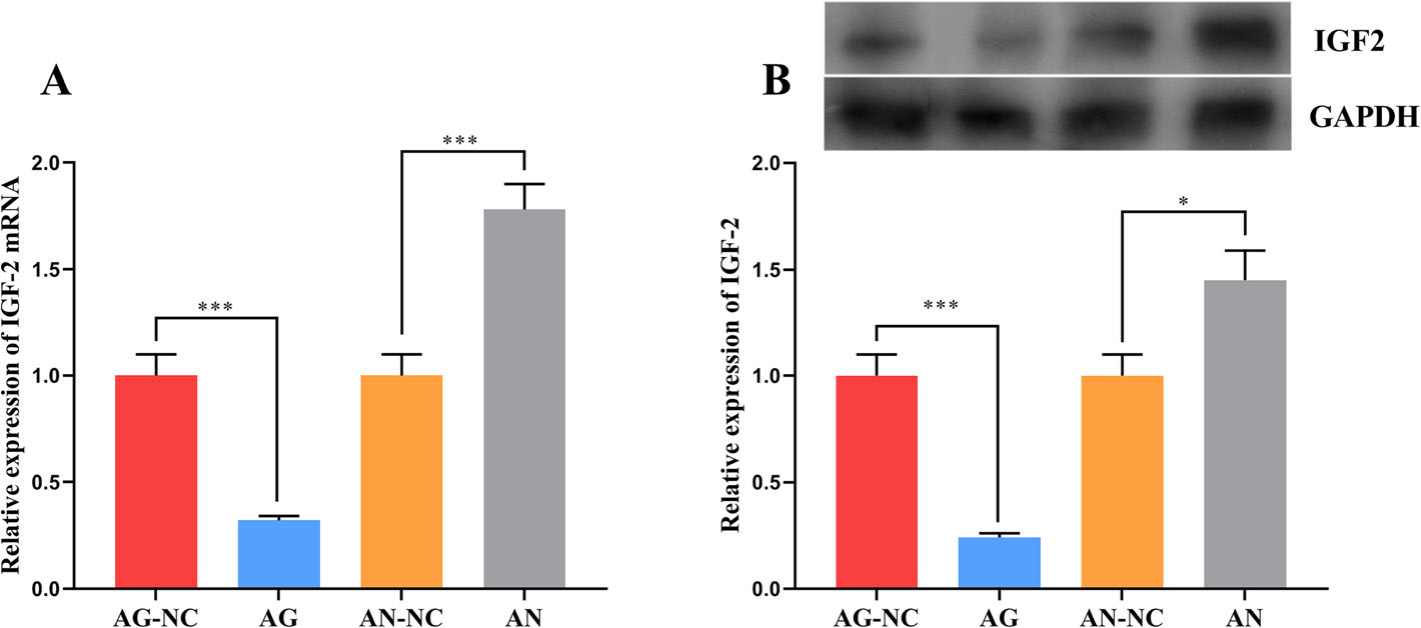
The effect of miR-637 in mice. **a** – Real-time PCR was done to detect changes in IGF-2 mRNA after miR-637 agomir or miR-637 antagomir were injected into the tail vein of the mice. **b** – Western blotting was performed to detect changes in IGF-2 after miR-637 agomir or miR-637 antagomir were injected into the tail vein of the mice. AG-NC: miR-637 agomir negative control; AG: miR-637 agomir; AN-NC: miR-637 antagomir negative control; AN: miR-637 antagomir. *** indicates *p* < 0.001

To verify the impact of miR-637 in a mouse model of atherosclerosis, we determined the expressions of IGF-2 mRNA and protein in the smooth muscle cells of the common carotid artery in the AG-NC, AG, AN-NC and AN groups. It was found that the expressions of IGF-2 mRNA and protein decreased after the injection of miR-637 agomir, while miR-637 antagomir led to the opposite results (Fig. 5a and b). We also measured the blood lipid content in mouse plasma, and found that the contents of TG, TC and LDL-C decreased significantly after the injection of miR-637 agomir, whereas the content of HDL-C increased significantly. Following the injection of miR-637 antagomir, the contents of the above substances showed the opposite changes (Table 2). We have confirmed that miR-637 restrains the proliferation and migration of VSMCs by targeting IGF-2 and affects the development of atherosclerosis.

**Table 2:** The effect of miR-637 on blood lipids in ApoE knockout mice ($\overline{\mathrm{x}}\kern0.5em \pm \kern0.5em \mathrm{s}$)

Group	AG-NC	AG	AN-NC	AN
Body weight (g)	26.48 ± 2.54	28.37 ± 2.88	27.52 ± 2.33	28.12 ± 2.42
TG (mmol/l)	2.46 ± 0.55	1.86 ± 0.52^&^	2.42 ± 0.62	3.82 ± 0.62***
TC (mmol/l)	14.78 ± 2.96	11.34 ± 2.21^&&^	15.32 ± 2.89	17.11 ± 3.82*
HDL-C (mmol/l)	3.44 ± 0.87	5.12 ± 0.95^&&&^	3.47 ± 0.78	2.35 ± 0.89*
LDL-C (mmol/l)	12.01 ± 1.12	10.12 ± 1.08^&&^	11.90 ± 1.32	14.78 ± 2.76**

*n* = 10; *AG-NC* miR-637 agomir negative control; *AG* miR-637 agomir, *AN-NC* miR-637 antagomir negative control, *AN* miR-637 antagomir, *TG* Triglyceride, *TC* Total cholesterol, *HDL-C* High density lipoprotein cholesterol, *LDL-C* Low density lipoprotein cholesterol

^&^, ^&&^ and ^&&&&^ respectively indicate *p* < 0.05, *p* < 0.01 and *p* < 0.001

*, ** and *** respectively indicate *p* < 0.05, *p* < 0.01 and *p* < 0.001

## Discussion

MiRNAs are abnormally expressed during the development of atherosclerosis, and some can promote or inhibit the proliferation and migration of VSMCs via regulation of downstream targets [24, 25]. Studies on the biological function of miRNA in atherosclerosis is of positive significance in finding new therapeutic targets.

In this study, we found that the expression of miR-637 was significantly downregulated in the plasma of patients with atherosclerosis, the plasma of ApoE^−/−^ C57BL/6 mice fed with a high-fat diet (a mouse model of atherosclerosis), and ox-LDL-treated VSMCs (a cell line model of atherosclerosis). Loss-of-function and gain-of-function experiments demonstrated that miR-637 represses the proliferation and migration of VSMCs. We also proved that the regulatory function of miR-637 in VSMCs is mediated by IGF-2.

In recent years, a variety of miRNAs have been shown to be involved in atherosclerosis, playing key roles in promoting or inhibiting the proliferation, migration and calcification of VSMCs. For instance, miR-205-5p targets the MICAL-2-regulated Erk1/2 signaling pathway to repress the proliferation of VSMCs [26]. After vascular injury, miR-451 restrains the migration of VSMCs via the Ywhaz/p38 MAPK pathway [27]. MiR-637 is abnormally expressed in multiple human diseases and is often thought to exert a regulatory effect on the proliferation, migration and other behaviors of various cells. For example, the expression of miR-637 decreases to facilitate the proliferation and migration of glioma cells [28]. MiR-637 blocks the migration of cholangiocarcinoma cells by interfering with CTSB [29].

In this study, the expression of miR-637 abnormally decreased in the plasma of atherosclerosis patients and ApoE^−/−^ mice fed with a high-fat diet. VSMCs were treated with ox-LDL to mimic atherosclerosis, and it was found that the expression of miR-637 varied depending on the concentration and treatment time. In addition, CCK-8 and Transwell assays demonstrated that transfection with miR-637 mimics suppressed the proliferation and migration of VSMCs, whereas miR-637 inhibitors had the opposite effect. Furthermore, after the mice were injected with miR-637 agonists and antagonists, the level of blood lipids in plasma was obviously changed. Based on these results, we conclude that miR-637 is one of the key factors in the occurrence and development of atherosclerosis.

IGF-2 is a growth factor with a complex regulatory pattern. Its activity is partially regulated by the differentially expressed IGF-2 receptor and IGF binding protein. IGF-2 has a vital role in cell growth and differentiation in diverse diseases through various signaling pathways [30]. For example, in lung cancer, IGF-2, which is regulated by miR-494, can facilitate the proliferation of A549 cells [31]. IGF-2 is one of the targets for miR-615-5p to modulate the proliferation of pancreatic ductal adenocarcinoma cells [32]. Moreover, IGF-2 is closely related to the proliferation of bovine retinal pigment epithelial cells and MCF-7 human breast cancer cells [33, 34].

Importantly, IGF-2 and its signaling receptors are crucial players in atherosclerosis. Neointimal hyperplasia after artery injury is partly regulated by the IGF axis, and IGFs stimulate VSMC proliferation and migration to form the neointima and upregulate tropoelastin synthesis after disruption of the elastic layer [35]. It is reported that targeted expression of IGF-2 in the VSMCs of mice leads to increased intimal thickenings [36].

In this study, IGF-2 was confirmed as a target of miR-637, and its expression level was shown to be inhibited by an miR-637 mimic. In VSMCs treated with ox-LDL, the trend of change for IGF-2 mRNA was the opposite of that for miR-637. Furthermore, transfection with miR-637 mimics impeded the expression of IGF-2 mRNA and protein. It was further verified that miR-637 could affect the expression of IGF-2 mRNA and protein in a mouse model with miR-637 agonists and antagonists. Thus, we conclude that miR-637 modulates the proliferation and migration of VSMCs by targeting IGF-2.

## Conclusion

Our in vitro and in vivo experiments show that miR-637 inhibits the proliferation and migration of VSMCs by downregulating IGF-2, thus affecting the progression of atherosclerosis. This study serves as an in-depth analysis of the molecular basis of atherosclerosis, and our findings should contribute to research into novel therapeutic targets.

## Data Availability

The data in this study are available from the author for correspondence upon reasonable request.

## Abbreviations

ApoE: Apolipoprotein E
CDNA: Complementary DNA
MiRNA: MicroRNA
Ox-LDL: Oxidized low-density lipoprotein
IGF-2: Insulin-like growth factor 2
VSMCs: Vascular smooth muscle cells
